# Predictors of early catheter replacement after HoLEP. Results from a high-volume laser center

**DOI:** 10.1590/S1677-5538.IBJU.2023.0165

**Published:** 2023-07-20

**Authors:** Fabrizio Di Maida, Anna Cadenar, Antonio Andrea Grosso, Luca Lambertini, Sofia Giudici, Daniele Paganelli, Vincenzo Salamone, Andrea Mari, Matteo Salvi, Andrea Minervini, Agostino Tuccio

**Affiliations:** 1 University of Florence Unit of Oncologic Minimally Invasive Urology and Andrology Department of Experimental and Clinical Medicine Florence Italy Department of Experimental and Clinical Medicine, University of Florence - Unit of Oncologic Minimally Invasive Urology and Andrology, Careggi Hospital, Florence, Italy

**Keywords:** Prostate, Catheters, Lasers, Solid-State, Transurethral Resection of Prostate

## Abstract

**Introduction:**

The aim of the study was to investigate clinical and surgical factors associated with early catheter replacement in patients treated with Holmium Laser Enucleation of the Prostate (HoLEP).

**Materials and Methods:**

Data of patients treated with HoLEP at our Institution by a single surgeon from March 2017 to January 2021 were collected. Preoperative variables, including non-invasive uroflowmetry and abdominal ultrasonography (US), were recorded. Bladder wall modifications (BWM) at preoperative US were defined as the presence of single or multiple bladder diverticula or bladder wall thickening ≥5 mm. Clinical symptoms were assessed using validated questionnaires. Only events occurred within the first week after catheter removal were considered.

**Results:**

Overall, 305 patients were included, of which 46 (15.1%) experienced early catheter replacement. Maintenance of anticoagulants/antiplatelets (AC/AP) therapy at surgery (p=0.001), indwelling urinary catheter (p=0.02) and the presence of BWM (p=0.001) were more frequently reported in patients needing postoperative re-catheterization. Intraoperative complications (p=0.02) and median lasing time (p=0.02) were significantly higher in this group. At univariate analysis, indwelling urinary catheter (p=0.02), BWM (p=0.01), ongoing AC/AP therapy (p=0.01) and intraoperative complications (p=0.01) were significantly associated with early catheter replacement. At multivariate analysis, indwelling urinary catheter (OR: 1.28; p=0.02), BWM (OR: 2.87; p=0.001), and AC/AP therapy (OR: 2.21; p=0.01) were confirmed as independent predictors of catheter replacement.

**Conclusions:**

In our experience the presence of indwelling urinary catheter before surgery, BWM and the maintenance of AC/AP therapy were shown to be independent predictors of early catheter replacement after HoLEP.

## INTRODUCTION

Latest European Guidelines on non-neurogenic male LUTS include Holmium Laser Enucleation of the Prostate (HoLEP) among treatment options for large benign prostatic hyperplasia (BPH). When compared to transurethral resection of prostate (TURP), HoLEP demonstrated similar long-term safety and efficacy, while being characterized by a slightly more favorable perioperative profile (1) Indeed, several studies reported that HoLEP is generally associated with shorter hospitalization and catheterization times, as well as lower transfusion and retreatment rates as compared to standard TURP (2, 3). Recent literature outlined similar findings in elderly and highly comorbid patients, thus making HoLEP a safe and valuable treatment option even in such subgroups (4, 5).

Despite the good perioperative profile, some patients still experience postoperative acute urinary retention (AUR) after catheter removal, thus leading to prolonged hospitalization, longer indwelling catheter time and higher readmission rates. However, to date only little evidence is available on perioperative variables associated with AUR following HoLEP (6, 7).

We hypothesized that several patient- and surgery-related features may act as adverse competing factors in inducing postoperative AUR. To fill this gap, in the present study we retrospectively reviewed our data aiming to investigate clinical and surgical predictors of early catheter replacement in patients treated with HoLEP for BPH.

## MATERIALS AND METHODS

### Patient dataset

After Institutional Review Board approval, clinical and surgical data of patients treated with HoLEP at our center from March 2017 to January 2021 were collected. The study was conducted in accordance with the ethical principles of the Declaration of Helsinki and all patients signed a written informed consent before enrollment.

**Figure 1:**
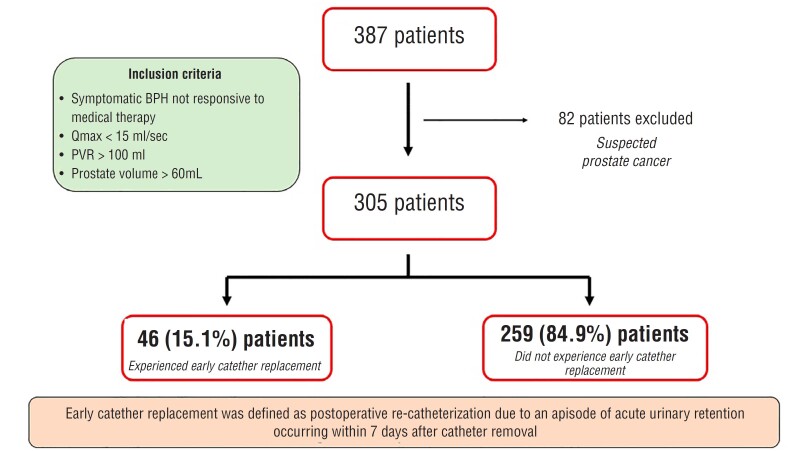
Flow chart depicting the study design.

Main inclusion criteria were: 1) symptomatic BPH not responsive to medical therapy, according to EAU guidelines (1); 2) Preoperative max flow rate (Qmax) at uroflowmetry < 15 mL/sec and/or post-voiding residual (PVR) > 100 mL; 3) Prostate volume > 60 mL. Patients with a prostate specific antigen (PSA) ≥ 4 ng/mL or suspect rectal examination underwent multiparametric magnetic resonance imaging (mpMRI) to rule out prostate cancer. Those with persistent suspect of prostate cancer were excluded from the study (Figure-1). All patients included in the study underwent preoperative non-invasive uroflowmetry with PVR examination and abdominal ultrasound (US) to assess prostate volume, presence of median lobe or bladder stones and bladder wall modifications (BWM), defined as the presence of either single or multiple bladder diverticula or bladder wall thickening ≥ 5 mm (1, 8).

Surgical-related variables included enucleation technique, overall operative time, enucleation time, morcellation time, lasing time, energy delivered and intraoperative complications. Since HoLEP relies on the contemporary use of laser and pulling movements, to be more accurate in quantifying the amount of energy delivered, we decided to separately report lasing and enucleation time. Particularly, enucleation time was defined as the time needed to enucleate the prostatic adenoma with both laser energy delivery and gentle mechanic traction, while lasing time referred to energy delivered for both enucleation and hemostasis. Overall surgical time included enucleation, morcellation and hemostasis time. Early and delayed postoperative complications were defined as any event occurring ≤30th or > 30th postoperative day, respectively, altering the normal postoperative course and/or delaying discharge. Postoperative complications were graded according to Clavien-Dindo classification.

A standard preoperative antibiotic regimen with Piperacillin-Tazobactam 4.5 gr was administered to all patients. In case of allergies, the alternative antibiotic therapy was Vancomycin 15 mg/Kg iv + Gentamicin 5 mg/Kg iv.

No special protocol was applied for patients taking AP/AC therapy. In case of suspension of coumadin, this was replaced with low molecular weight heparin (LMWH) 5 days before the procedure, while a suspension period starting from 48 hours before the procedure was generally applied for novel oral anticoagulants. The LMWH was therefore continued postoperatively before reintroducing AC therapy for a variable period defined by the anesthesiologists in relation to the individual risk profile. In case of AP therapy, a LMWH with prophylactic dose was routinely applied as in any other endoscopic surgery.

### Surgical technique

**Figure 2:**
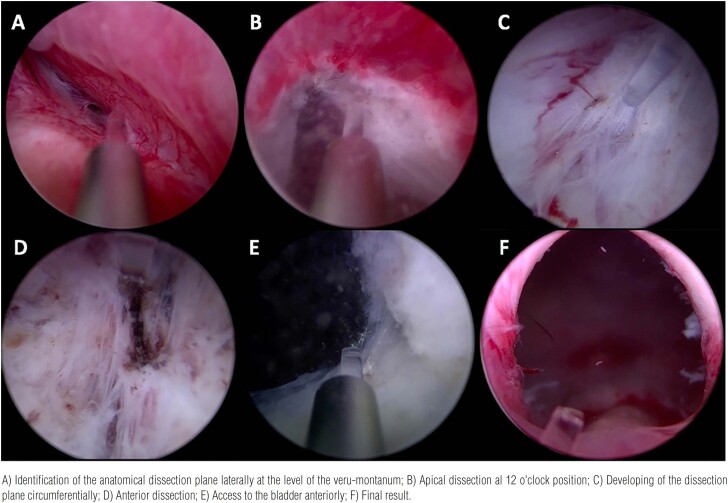
HoLEP surgical steps.

All procedures were performed by a single surgeon, and enucleation was conducted according to operator’s preference with either the three-lobes or the ^en-bloc^ with early apical release technique, as described in previous investigations (Figure-2) (9, 10).

All procedures were carried out under general anesthesia using the 120W Versapulse holmium laser machine (Lumenis, Yokneam, Israel) with a 550-µm end laser fiber (Boston Scientific, AccuMax 550 Laser Fiber). Laser energy was set at 2 J X 45 Hz, 90 W, for enucleation and 2 J X 30 Hz, 60 W, for coagulation. A 26F Storz continuous flow resectoscope sheath was modified by inserting the 26F inner sheath, and a laser bridge to stabilize the fiber. A 30° down lens was preferred. The enucleated prostatic adenoma was then morcellated using a morcellator (Lumenis, Versacut). After surgery, a 22F three-way catheter was inserted and bladder irrigation was performed using saline solution. We usually removed urethral catheter on 3rd postoperative day, in case of clear urine output.

### Outcome measures and follow-up

Assessment visits, including uroflowmetry and PVR determination by abdominal US, were scheduled at screening visit on day 0 and then at 3 and 12-month follow up after the surgical intervention. Clinical evaluation was assessed using the Italian version of the following validated questionnaires: IPSS (international prostate symptom score) (11), OAB-q SF (Overactive Bladder Questionnaire-Short Form) (12), ICIQ-SF (International Consultation on Incontinence Questionnaire-Urinary Incontinence Short Form) (13) and the IIEF-5 (international index of erectile function) (14).

### Endpoints

Patients were divided into two groups according to early catheter replacement, defined as postoperative re-catheterization due to an episode of AUR that occurred within 7 days after catheter removal. The primary endpoint of our study was to apprise any difference between the two groups in terms of perioperative and/or surgical variables.

### Statistical Analysis

Continuous variables are presented as median (IQR: interquartile range) and differences between groups were tested by Student’s independent t-test or Mann-Whitney U-test according to their normal or not-normal distribution, respectively (normality of variables’ distribution was tested by Kolmogorov-Smirnov test). Proportional data were assessed using the Chi-square test. To assess clinical differences from baseline to follow-up the median change and test for non-parametric differences were applied. All tests were two-sided. Statistical significance was set as p <0.05. Statistical analysis was performed using SPSS v. 27 (IBM SPSS Statistics for Mac, Armonk, NY, IBM Corp).

## RESULTS

Overall, 305 patients were included in our study and stratified into two groups according to postoperative re-catheterization. Forty-six (15.1%) experienced early catheter replacement due to an episode of AUR. Particularly, 9 (19.6%) patients underwent postoperative re-catheterization within the first 24 hours after catheter removal. Preoperative characteristics are reported in Table-1. Particularly, patients needing catheter replacement showed a significantly higher use of anticoagulant (AC) or antiplatelet (AP) medications at surgery (11.5% vs 41.3%, p = 0.001) and a higher rate of BWM (6.9% vs 19.5%, p = 0.001), as well as a higher rate of indwelling urinary catheter before surgery (14.2% vs 34.7%, p = 0.02).

**Table 1:** Preoperative characteristics of 305 patients treated with Holmium Laser Enucleation of the Prostate (HoLEP).

Variables		Group A (n=259; 84.9%)	Group B (n=46; 15.1%)	p-value
Preoperative characteristics				
Age (years) (median, IQR)		69 (65 - 74)	70 (64 – 75)	0.13
BMI (kg/m^2^) (median, IQR)		26 (23.7 – 28.1)	26.1 (24.4 – 28.5)	0.73
CCI age adjusted (median, IQR)		3 (1 – 4)	3 (1 – 4)	0.43
ASA score (median, IQR)		2 (1 - 3)	2 (1 - 3)	0.21
ACs/APs therapy at surgery (n, %)		30 (11.5)	19 (41.3)	0.001
Bladder stone (n, %)		34 (13.1)	7 (15.2)	0.45
Median prostate lobe (n, %)		101 (38.9)	19 (41.3)	0.23
BPH therapy (n, %)	Alpha-blockers	136 (52.5)	27 (58.6)	0.30
	5-ARIs	41 (15.8)	9 (19.5)	
	Combination therapy	56 (21.6)	10 (21.7)	
AUR (n, %)	Overall	113 (43.6)	25 (54.3)	0.10
	Single/multiple episodes	76 (29.3)	11 (23.9)	
Indwelling catheter (n, %)		37 (14.2)	16 (34.7)	0.02
BWM (n, %)		18 (6.9)	9 (19.5)	0.001
Prostate volume (mL) (median, IQR)		100 (76 – 130)	109 (76 – 130)	0.39
Creatinine serum level (mg/dL) (median, IQR)		1.0 (0.9-1.2)	0.9 (0.9-1.1)	0.91
Hb blood level (g/dL) (median, IQR)		14 (13.1-15.2)	14.9 (13.7-15.3)	0.34
Q-max (mL/s) (median, IQR)		8.2 (7.0 – 10.0)	8.7 (7.3 – 10.3)	0.47
PVR volume (mL) (median, IQR)		160 (100 – 250)	150 (100 – 280)	0.17
PSA serum level (ng/mL) (median, IQR)		5.6 (2.5 – 7.3)	4.8 (2.8 – 8.7)	0.25
IPSS score (median, IQR)		24 (21 – 28)	24 (21 – 27)	0.63
IIEF-5 score (median, IQR)		18 (12 – 22)	18 (10 – 21)	0.70
OAB-q score (median, IQR)		44 (25 – 55)	39 (27 – 53)	0.76
ICIQ-sf score (median, IQR)		0 (0 – 0)	0 (0 – 0)	0.42
QoL score (median, IQR)		4 (3 – 5)	4 (4 – 5)	0.34

Group A = catheter-free patients; Group B = patients experiencing early catheter replacement.

AC = Anticoagulants; AP = Antiplatelets; ASA = American Society of Anesthesiologists; AUR = Acute Urinary Retention; BMI = Body mass index; BPH = Benign Prostatic Hyperplasia; BWM = Bladder Wall Modification; CCI = Charlson Comorbidity Index; HB = Hemoglobin; ICIQ-q = International Consultation on Incontinence Modular questionnaire; IIEF-5 = International Index of Erectile Function; IPSS = International Prostate Symptom Score; OAB-q = Overactive Bladder questionnaire; PVR = Post-voiding residual; QoL = Quality of Life

Intraoperative and surgical features are described in Table-2. Patients experiencing re-catheterization presented a longer median lasing time (30 min [IQR 29 - 40] vs 38 min [IQR 29 - 48], p = 0.02), while enucleation and morcellation time, total energy delivered during HoLEP and enucleation technique were comparable between the two cohorts. Moreover, patients undergoing early catheter replacement reported a higher percentage of intraoperative complications (8.8% vs 13%, p = 0.02), including capsule perforation and/or bladder mucosal damage.

**Table 2:** Intraoperative features and surgical outcomes of 305 patients treated with Holmium Laser Enucleation of the Prostate (HoLEP).

Variables		Group A (n=259; 84.9%)	Group B (n=46; 15.1%)	p-value
Surgical Outcomes				
Enucleation Technique (n, %)	Three-lobes	105 (40.5)	22 (47.8)	0.17
	En-bloc	154 (59.5)	24 (52.2)	
Overall operative time (min) (median, IQR)		100 (67 – 120)	97 (65 – 115)	0.23
Enucleation time (min) (median, IQR)		52 (35 – 60)	45 (32 – 55)	0.24
Morcellation time (min) (median, IQR)		24 (16 – 35)	23 (16 – 32)	0.17
Lasing time (min) (median, IQR)		30 (29 – 40)	38 (29 – 48)	0.02
Energy delivered (kJ) (median, IQR)		120.1 (100.9 – 140.3)	131.3 (103.2 – 162.6)	0.48
Intraoperative complication, (n, %)		23 (8.8)	6 (13.0)	0.02
Capsule perforation		14 (5.4)	5 (10.8)	
Bladder mucosal damage		9 (3.4)	1 (2.2)	
Surgical Era (n, %)	≤ 50 procedures	41 (15.8)	9 (19.5)	0.39
	> 50 procedures	218 (84.2)	37 (80.5)	

Group A = catheter-free patients; Group B = patients experiencing early catheter replacement.

As regards postoperative variables (Table-3), patients experiencing early catheter replacement showed a significantly longer hospitalization compared to catheter-free patients (4 days [IQR 3 - 5] vs 6 days [IQR 4 - 7], p=0.001). Early and delayed postoperative surgical complication rates were comparable between the two cohorts (p=0.21). At 3-month assessment, median PSA, Q-max, urinary incontinence and clinical symptoms assessed by dedicated questionnaires did not significantly differ in the two study groups (all p > 0.05). On the contrary, median PVR appeared lower in catheter-free patients (30 mL [IQR 8 - 50] vs 60 [IQR 40 - 100], p = 0.02). Clinical assessment at 12-month follow-up did not reveal any significant differences between the two groups in terms of patient reported outcomes, with also difference in PVR mitigating between the two groups (35 mL [10 - 55] vs 55 [40 - 80], p = 0.12).

**Table 3:** Postoperative, functional and self-reported outcomes of 305 patients treated with Holmium Laser Enucleation of the Prostate (HoLEP).

Variables		Group A (n=259; 84.9%)	Group B (n=46; 15.1%)	p-value
Postoperative Outcomes				
Hospitalization time (days) (median, IQR)		4 (3 – 5)	6 (4 – 7)	0.001
Early events	36 (13.8)	8 (17.3)	0.21		
CD≤2	32 (12.3)	7 (15.2)			
CD>2	4 (1.5)	1 (2.1)			
Delayed events	4 (1.5)	1 (2.1)			
CD≤2	1 (0.3)	1 (2.1)			
CD>2	3 (1.2)	0 (0)			
Follow-up (month) (median, IQR)		18 (9-29)	17 (9-27)	0.19
Functional results and PROMs at 3-month follow up				
UI (n, %)		19 (7.3)	3 (6.5)	0.32
Q-max (mL/s) (median, IQR)		23 (17 – 27)	21 (17 – 26)	0.26
PVR volume (mL) (median, IQR)		30 (8 – 50)	60 (40 – 100)	0.02
PSA (ng/mL) (median, IQR)		0.9 (0.63 – 1.00)	0.9 (0.68 – 1.60)	0.17
IPSS (median, IQR)		9 (2 – 12)	6 (1 – 8)	0.19
IIEF-5 (median, IQR)		17 (12 – 20)	18 (11 – 20)	0.81
OAB-q (median, IQR)		15 (13 – 19)	13 (13 – 16)	0.06
ICIQ-sf (median, IQR)		1 (0 – 2)	0 (0 – 0)	0.08
QoL (median, IQR)		1 (0 – 2)	1 (0 – 1)	0.13
Functional results and PROMs at 12-month follow up				
UI (n, %)		7 (2.7)	0 (0.0)	0.29
Q-max (mL/s) (median, IQR)		22 (15 – 26)	21 (16 – 25)	0.34
PVR volume (mL) (median, IQR)		35 (10 – 55)	55 (40 – 80)	0.12
IPSS (median, IQR)		10 (2 – 11)	7 (1 – 9)	0.21
IIEF-5 (median, IQR)		16 (11 – 19)	18 (11 – 20)	0.78
OAB-q (median, IQR)		16 (12 – 18)	12 (10 – 16)	0.12
ICIQ-sf (median, IQR)		1 (0 – 2)	0 (0 – 0)	0.08
QoL (median, IQR)		1 (0 – 2)	1 (0 – 1)	0.13

At univariate analysis, indwelling urinary catheter before surgery (p=0.02), BWM (p=0.01), ongoing AC/AP therapy (p=0.01) and intraoperative complications (p=0.01) were significantly associated with early catheter replacement. At multivariate analysis, indwelling urinary catheter (OR: 1.28; CI 95%: 1.21 – 2.11 p = 0.02), BWM (OR:2.87; CI 95%:1.25-3.26; p=0.001) and AC/AP therapy (OR:2.21; CI 95%: 1.10-2.31; p=0.01) were confirmed as independent predictors of catheter replacement after HOLEP (Table-4).

**Table 4:** Univariate and Multivariate logistic regression analysis for the predictors of Early catheter replacement.

Variates	Univariate analysis		Multivariate analysis	
	OR (95% CI)	p-value	OR (95% CI)	p-value
Indwelling catheter	1.32 (1.18-2.24)	**0.02**	1.28 (1.21-2.11)	**0.02**
Bladder wall modifications	2.51 (1.29-3.71)	**0.01**	2.87 (1.25-3.26)	**0.001**
On-going ACs/APs at surgery	2.23 (1.11-2.33)	**0.01**	2.21 (1.10-2.31)	**0.01**
Intraoperative complication	1.54 (1.45-2.11)	**0.01**	1.21 (0.94-2.13)	0.09
Lasing time (continuous variable)	1.11 (0.27-1.84)	0.53	–	–

AC = Anticoagulant; AP = Antiplatelet.

## DISCUSSION

While current available literature reports plenty of evidence investigating the safety and efficacy of different techniques for the surgical management of BPH (15), there is far less investigation into the HoLEP field in the setting of predictors of early catheter replacement. Indeed, although the surgical technique has already reached a high standardization, we still need finer tools to timely identify those patients eventually experiencing an early failure in resuming normal micturition after surgery. To address this unmet need, in the current paper we sought to analyze our high-volume single institutional series seeking for any clinical or surgical predictors of early catheter replacement. AUR after catheter removal was recorded in nearly 15% of cases and was associated either with blood clots or bladder neck spasm / postoperative oedema. Our data are consistent with current literature, since postoperative AUR was reported to range between 0% and 16% in previously published studies (16–18). Notably, we demonstrated that indwelling urinary catheter before surgery, AC/AP and BWM were independent predictors of early AUR after HoLEP, thus further highlighting three additional features worth of discussion at the time of preoperative counselling.

First key finding of our study is that the occurrence of AUR after catheter removal was not associated to the learning curve nor enucleation technique used, being no significant differences demonstrable between patients treated with “^en-bloc^” and “^three-lobes^” enucleation. Interestingly, median lasing time was significantly higher in those patients experiencing early catheter replacement, although the latter was not confirmed as an independent predictor of postoperative AUR at multivariable analysis. A longer lasing time could in fact hardly justify a greater risk of AUR, but may rather reflect a higher attention in hemostasis, since patients needing catheter replacement showed a significantly higher use of AC/AP at baseline. The hypothetical risks carried by the ongoing AC/AP therapy during HoLEP was already confirmed by different previous studies (6, 19, 20), although it should be highlighted that the rate of postoperative bleeding even in case of AC/AP therapy is anyhow quite acceptable and makes HoLEP an excellent technique to treat also complex patients. In this regard, our group recently published a paper demonstrating that, in experienced hands, HoLEP represents an effective option for the treatment of BPH also for high comorbid patients (5). The observed benefit of HoLEP in maintaining hemostasis in AC/AP patients is likely due to the physics of the holmium laser (21). Indeed, due to the chromophore of water and minimal tissue depth penetration, holmium laser is able to achieve quick vaporization and coagulation of tissue without the disadvantage of deep tissue penetration. The safety of the surgical technique is further bolstered by the consideration that early and delayed postoperative complications did not differ between the two cohorts in our study, although a non-significant trend was observed in patients experiencing postoperative AUR.

Second key finding of the study is related to the influence of BWM in determining the resume of normal micturition after surgery. Indeed, BWM was the strongest independent predictor of AUR after HoLEP. To the best of our knowledge this is the first report correlating BWMs to risk for early catheter replacement after HoLEP. Indeed, bladder wall thickness had already been associated with risk of AUR before surgical management of obstructive BPH (22). BWM has been correlated with detrusor function. In this regard, Oelke et al. found that detrusor wall thickness increases depending on the extent of bladder outlet obstruction (23). Therefore, measurement of bladder wall thickness has been proposed as a useful and simpler diagnostic parameter as it could act as a possible marker to replace conventional urodynamic pressure flow study in patients with bladder outlet obstruction (24). Indeed, as hypertrophy of the bladder musculature advances, there is an increase in the collagen component, gradually replacing the muscular fibers. The changes are coupled with a relative ischemia of the hypertrophic muscle fibers, being muscle hypertrophy not supported by a proportional neoangiogenesis. As a result, the increase in interstitial collagen reduces the distensibility of the bladder with consequent rise in intravesical pressure and leads to a progressive reduction in contractility of the detrusor. In this regard, in our experience indwelling urinary catheter before surgery was confirmed as an independent predictor of early catheter replacement after HoLEP, thus further highlighting the residual preoperative bladder contractility as a main key driver of resume of normal micturition after surgery.

Interestingly, in our study patients experiencing ^de novo^ catheter replacement still had a significantly higher PVR at 3-month evaluation, as compared to their counterpart. However, such difference was not statistically significant at 12-month assessment. Of note, our group first introduced the concept of “trifecta” in HoLEP (25). Multivariate analysis confirmed PVR ≥ 250 mL as one of the independent predictors of Trifecta failure, further highlighting the decompensation of the detrusor as one the main key driver of postoperative outcomes. Based on these findings, it may be reasonable to offer urodynamic study to patients preoperatively presenting with BWM, as we could speculate those individuals could experience higher difficulties in resuming normal micturition after catheter removal. Nonetheless, the role of BPH surgery in case of concomitant detrusor underactivity remains controversial (26). Indeed, it is also true that in this context pressure/flow study would only add the benefit to better forecast the room for improvement after BPH surgical management, thus further tailoring preoperative counselling, as it would hardly change the choice to relieve prostatic obstruction.

The present paper is not devoid of several limitations. This was a retrospective review of a prospectively collected database, thus the study design might have weakened itself the reliability of evidence reported. Second, all cases were performed by a single highly trained surgeon with an extensive experience in endoscopic surgery. As such, our findings could not be applicable to all surgeon- or center-related scenarios.

Despite of these limitations, the findings of the current series represent one of the largest series exploring predictors of ^de novo^ catheterization after HoLEP. Further studies with longer follow-up are eagerly warranted to validate the preliminary findings of the current series.

## CONCLUSIONS

Indwelling urinary catheter before surgery, bladder wall modifications and maintenance of anticoagulant/antiplatelet therapy were shown to be independent predictors of early catheter replacement after HoLEP. Such features should be carefully discussed with patients at the time of preoperative counselling as they could eventually impair surgical outcomes after bladder outlet obstruction relief.
